# ER-phagy mediates selective degradation of endoplasmic reticulum independently of the core autophagy machinery

**DOI:** 10.1242/jcs.154716

**Published:** 2014-09-15

**Authors:** Sebastian Schuck, Ciara M. Gallagher, Peter Walter

**Affiliations:** Howard Hughes Medical Institute and Department of Biochemistry and Biophysics, University of California San Francisco, 600 16th Street, San Francisco, CA 94158, USA

**Keywords:** Endoplasmic reticulum, Stress response, Autophagy

## Abstract

Selective autophagy of damaged or redundant organelles is an important mechanism for maintaining cell homeostasis. We found previously that endoplasmic reticulum (ER) stress in the yeast *Saccharomyces cerevisiae* causes massive ER expansion and triggers the formation of large ER whorls. Here, we show that stress-induced ER whorls are selectively taken up into the vacuole, the yeast lysosome, by a process termed ER-phagy. Import into the vacuole does not involve autophagosomes but occurs through invagination of the vacuolar membrane, indicating that ER-phagy is topologically equivalent to microautophagy. Even so, ER-phagy requires neither the core autophagy machinery nor several other proteins specifically implicated in microautophagy. Thus, autophagy of ER whorls represents a distinct type of organelle-selective autophagy. Finally, we provide evidence that ER-phagy degrades excess ER membrane, suggesting that it contributes to cell homeostasis by controlling organelle size.

## INTRODUCTION

Autophagy is the transport of cytoplasmic constituents into lysosomes. Cells utilize this versatile process for many purposes, including the non-selective breakdown of cytoplasm during starvation and the selective removal of damaged or redundant organelles. Three principal types of autophagy are currently being distinguished, namely macroautophagy, microautophagy and, in mammals, chaperone-mediated autophagy (Klionsky, 2004).

The key characteristic of macroautophagy is the generation of autophagosomes, which are large double-membrane transport vesicles that engulf their cargo as they form. Autophagosomes subsequently fuse with lysosomes and release their contents into the lytic compartment as part of single-membrane autophagic bodies. Many autophagy-related (Atg) proteins have been identified and can be divided into two groups. The well-conserved core autophagy machinery is essential for the assembly of all autophagosomes and, in yeast, comprises fifteen proteins (Atg1–Atg10, Atg12–Atg14, Atg16 and Atg18). The remaining Atg proteins function as accessory factors and determine whether cargo engulfment is non-selective or selective (Xie and Klionsky, 2007; Mizushima et al., 2011).

Microautophagy is much less well understood than macroautophagy. In its current use, the term refers to a collection of diverse processes. The defining feature of microautophagy is that cytoplasmic constituents are taken up into lysosomes by invagination and inward budding of the lysosomal membrane. In both yeast and mammals, microautophagy can mediate the uptake of small portions of cytoplasm (Müller et al., 2000; Mijaljica et al., 2011). This can occur without a functional core autophagy machinery (Sattler and Mayer, 2000; Sahu et al., 2011). In addition, several autophagic processes exist that selectively target particular organelles and likewise involve cargo engulfment by the lysosomal membrane. In yeast, this has been observed for peroxisomes, mitochondria, lipid droplets and parts of the nucleus (Kiššová et al., 2007; Kvam and Goldfarb, 2007; Manjithaya et al., 2010; van Zutphen et al., 2014). Although these types of organelle-selective autophagy do not involve autophagosomes, they all require the core autophagy machinery, likely for the final closure of microautophagic invaginations of the vacuolar membrane (Strømhaug et al., 2001; Mukaiyama et al., 2002; Mukaiyama et al., 2004; Kiššová et al., 2007; Krick et al., 2008; van Zutphen et al., 2014). Further mechanistic information remains scarce, however, and only a handful of other proteins have been implicated in these processes (Roberts et al., 2003; Dubouloz et al., 2005; Uttenweiler et al., 2007). Moreover, the different types of organelle-selective autophagy are morphologically distinct. In yeast, microautophagy of organelle-free cytoplasm proceeds through deeply invaginated autophagic tubes that release small vesicles at their tips, microautophagy of peroxisomes involves a specialized membrane structure that mediates the closure of arm-like extensions of the lysosomal membrane, and microautophagy of the nucleus is initiated at pre-existing contact sites between the nucleus and the lysosome (Müller et al., 2000; Roberts et al., 2003; Mukaiyama et al., 2004). Thus, the relationship between the various types of autophagy subsumed under the term microautophagy remains to be established. In addition, some of these processes involve engulfment of large cargoes and in these cases the descriptor ‘micro’ appears to be a misnomer.

The endoplasmic reticulum (ER) is linked to autophagy in several ways. First, ER stress, which is caused by the accumulation of misfolded proteins in the ER lumen, triggers macroautophagy in both yeast and mammals (Bernales et al., 2006; Ogata et al., 2006; Yorimitsu et al., 2006). Second, many autophagosomes form at the ER (Axe et al., 2008; Hayashi-Nishino et al., 2009; Ylä-Anttila et al., 2009; Matsunaga et al., 2010; Suzuki et al., 2013; Graef et al., 2013). Third, the ER itself can become subject to autophagy. In yeast, starvation-induced autophagosomes include short ER fragments (Hamasaki et al., 2005). It is unclear if this represents selective autophagy or is a consequence of how autophagosomes form (Hayashi-Nishino et al., 2009). Accumulation of aggregation-prone proteins in the ER triggers their removal through macroautophagy, but the degree of selectivity of this process has not been determined (Kamimoto et al., 2006; Kruse et al., 2006; Fu and Sztul, 2009; Ishida et al., 2009; Lipatova et al., 2013). Selective autophagy of ER has been observed, however, during the formation of storage granules in the insect fat body and the degradation of expanded smooth ER in rat hepatocytes (Locke and Collins, 1965; Bolender and Weibel, 1973). The mechanisms underlying these cases of ER-selective autophagy are unknown.

We found previously that ER stress in the yeast *Saccharomyces cerevisiae* leads to massive ER expansion, which increases the protein folding capacity of the ER (Bernales et al., 2006; Schuck et al., 2009). Furthermore, ER stress can induce large ER whorls, which have been proposed to be subject to autophagy (Bernales et al., 2006). Here, we further dissect the cellular response to ER stress in yeast and show that it involves a distinct type of organelle-selective autophagy that does not require the known core autophagy machinery.

## RESULTS

### ER stress induces autophagy of ER whorls

**Fig. 1.**
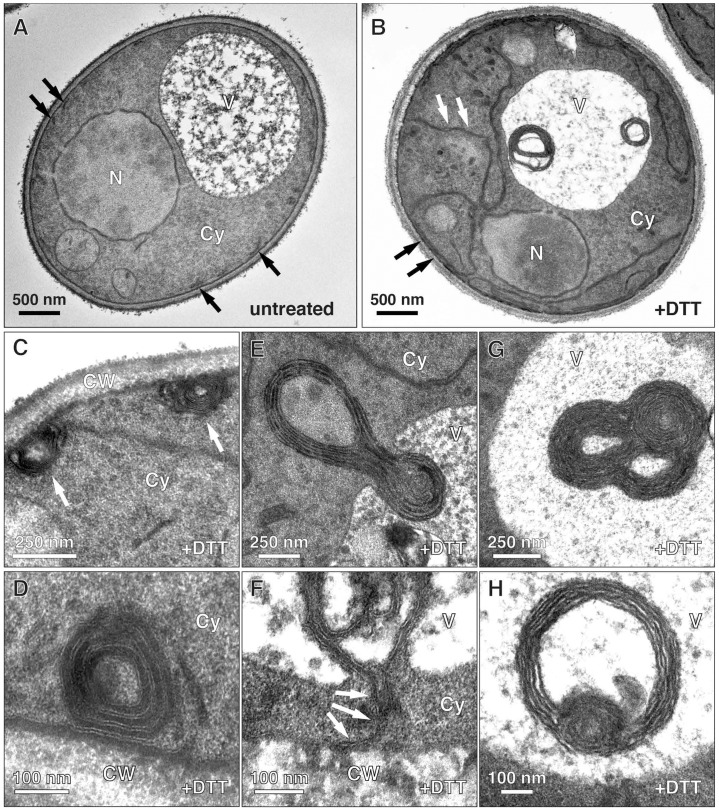
**ER stress induces autophagy of ER whorls.** (A) Electron micrograph of untreated wild-type yeast. Black arrows indicate cortical ER elements. (B) Wild-type yeast treated with DTT for 3 h. Note the expanded peripheral ER (black arrows), cytoplasmic ER extensions (white arrows) and ring-shaped ER whorls inside the vacuole. (C,D) ER whorls at the cell cortex. Micrographs are from cells treated with DTT for 1 h. (E,F) Engulfment of ER whorls by the vacuolar membrane. Micrographs are from cells treated with DTT for 4 h, but similar uptake intermediates were observed from 1 h onwards. In E, note the invagination of the vacuolar membrane during whorl uptake. In F, note the continuity between the peripheral ER and an ER whorl that is partially sequestered in a vacuolar membrane invagination (white arrows). (G,H) ER whorls in the vacuole. Micrographs are from cells treated with DTT for 3 h. CW, cell wall; Cy, cytoplasm; N, nucleus; V, vacuole.

We first employed electron microscopy to analyze ER morphogenesis during ER stress. In untreated yeast, the peripheral ER was visible as short membrane profiles underneath the plasma membrane (Fig. 1A, black arrows). When cells were treated with dithiothreitol (DTT) to block disulfide bond formation and induce protein misfolding in the ER, the peripheral ER appeared as long stretches of cortical membrane with prominent cytoplasmic extensions (Fig. 1B, black and white arrows, respectively). These profiles correspond to expanded membrane sheets (Schuck et al., 2009). In addition, we observed round, often ring-shaped membrane structures, which were most obvious in the vacuole (Fig. 1B). Their characteristic dark staining suggested that they were derived from the ER.

**Fig. 2.**
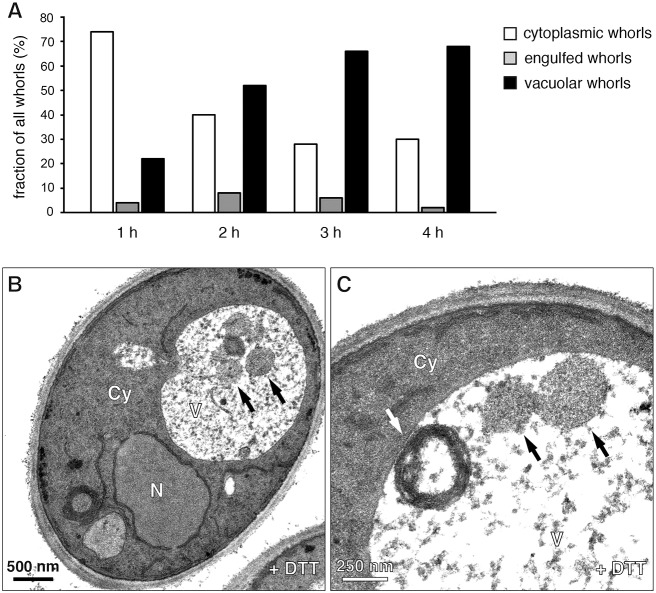
**ER stress activates both autophagy of ER whorls and non-selective macroautophagy.** (A) Quantification of ER whorls in the cytoplasm, ER whorls engulfed by vacuolar membrane and ER whorls inside the vacuole in wild-type yeast treated with DTT for up to 4 h. 50 whorls were counted per time point and the fraction of cytoplasmic, engulfed and vacuolar whorls was determined. (B,C) Electron micrographs of wild-type yeast treated with DTT for 4 h. In B, black arrows indicate autophagic bodies containing cytoplasm. C shows autophagic bodies (black arrows) and an ER whorl (white arrow) in the same vacuole. Cy, cytoplasm; N, nucleus; V, vacuole.

To better understand the generation and fate of these membrane whorls, we followed them over time. After 1 h of DTT treatment, ER whorls were predominantly seen underneath the plasma membrane and were 200 nm in diameter (Fig. 1C,D; see supplementary material Fig. S1A for serial sections). The ER was tightly packed in these multi-lamellar structures, with individual layers consisting of a dark stripe of ER lumen delimited by two white lines that correspond to the enclosing lipid bilayers (Fig. 1D). In addition, from 1 h of DTT treatment onwards, ER whorls were found in vacuolar membrane invaginations (Fig. 1E,F). Virtually no cytoplasm was visible between an ER whorl and the enveloping vacuolar membrane, suggesting that engulfment is highly selective. Occasionally, ER whorls in vacuolar membrane invaginations were still connected to the cell cortex (Fig. 1F, white arrows; see supplementary material Fig. S1B for serial sections), indicating that whorl formation and sequestration can take place concurrently and confirming that the membrane whorls derive from the peripheral ER. By 3 h of DTT treatment, most ER whorls were inside the vacuole and had diameters of 300–800 nm (Fig. 1G,H). ER whorls at the cell periphery and inside the vacuole typically appeared as rings in single sections, but serial sections revealed that they were hollow spheres (supplementary material Fig. S1A,C). Furthermore, serial sections showed that whorls in the vacuole were completely disconnected from the surrounding cytoplasm (supplementary material Fig. S1C). Quantification revealed that 75% of whorls where cytoplasmic after 1 h of DTT treatment, ∼5% were engulfed by vacuolar membrane and thus likely in the process of being taken up into the vacuole, and 20% were already inside the vacuole. Over time, the fraction of cytoplasmic whorls dropped to 30%, engulfed whorls remained rare and the fraction of whorls in the vacuole increased to over 65% (Fig. 2A).

Taken together, these observations indicate that ER whorls derive from the peripheral ER and are imported into the vacuole as part of an autophagic process. The vacuolar membrane invaginations seen in uptake intermediates indicate a mechanism topologically equivalent to microautophagy. We established that tunicamycin, an ER stressor that inhibits protein N-glycosylation, also induced the formation of ER whorls as well as their delivery into the vacuole by invagination and inward budding of the vacuolar membrane (supplementary material Fig. S2). This finding suggests that autophagy of ER whorls is a general response to strong ER stress. Finally, we found that autophagic bodies containing cytoplasm accumulated in the vacuole after prolonged DTT treatment (Fig. 2B). After 4 h, over 40% of vacuole-localized ER whorls were contained within vacuoles that showed autophagic bodies in the same thin section (15 out of 34 counted, see Fig. 2C for an example), indicating that ER stress can activate both selective autophagy of ER whorls and non-selective macroautophagy in the same cell.

**Fig. 3.**
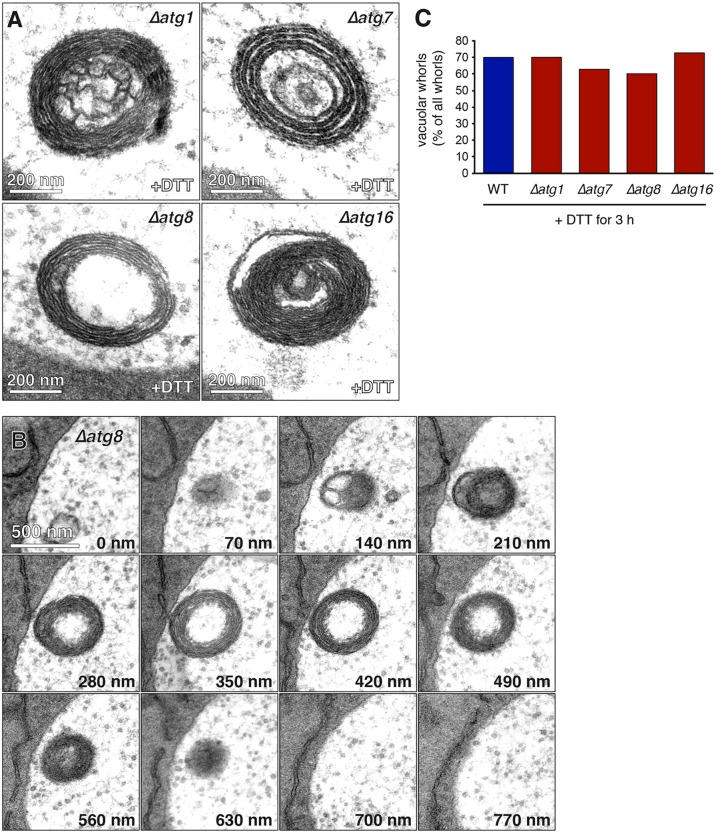
**Autophagy of ER whorls does not require the core autophagy machinery.** (A) Electron micrographs of ER whorls in the vacuoles of *Δatg1*, *Δatg7*, *Δatg8* and *Δatg16* cells treated with DTT for 3 h. (B) Electron micrographs of serial 70-nm thin sections showing an ER whorl that has been taken up into the vacuole of a *Δatg8* cell treated with DTT for 3 h. (C) Quantification of ER whorls in the vacuole in cells treated with DTT for 3 h. 30 whorls were counted per strain and the fraction of whorls in the vacuole was determined. Whorls engulfed by the vacuolar membrane were counted as cytoplasmic whorls.

Given the central importance of the core autophagy machinery for the various types of selective autophagy known so far, we tested whether it was required for autophagy of ER whorls. When mutant yeast lacking Atg1, Atg7, Atg8 or Atg16 were treated with DTT, ER whorls were still generated and imported into the vacuole (Fig. 3A, see also supplementary material Fig. S3A). The same was true for yeast lacking Atg11, which is not part of the core autophagy machinery but is an essential cargo adaptor for various types of selective autophagy (data not shown). There were no obvious differences in whorl morphology compared with wild-type cells, and serial sections confirmed that the mutants were able to complete the import of ER whorls into the vacuole (Fig. 3B; supplementary material Fig. S3B). Quantification showed that the fraction of whorls that had already been imported into vacuoles after 3 h of DTT treatment was 60–70% in both wild-type and *atg1*, *atg7*, *atg8* and *atg16* mutant cells, indicating that the efficiency of the process was essentially unaffected by deletion of key *ATG* genes (Fig. 3C). Thus, autophagy of ER whorls does not require the core autophagy machinery, which sets it apart from all other previously described types of organelle-selective autophagy.

To complement our morphological analysis, we next followed autophagy biochemically. We thereby sought to test the conclusions reached so far, namely that ER stress activates distinct modes of autophagy, including selective autophagy of ER that is independent of the core autophagy machinery.

### ER stress triggers a composite autophagic response

**Fig. 4.**
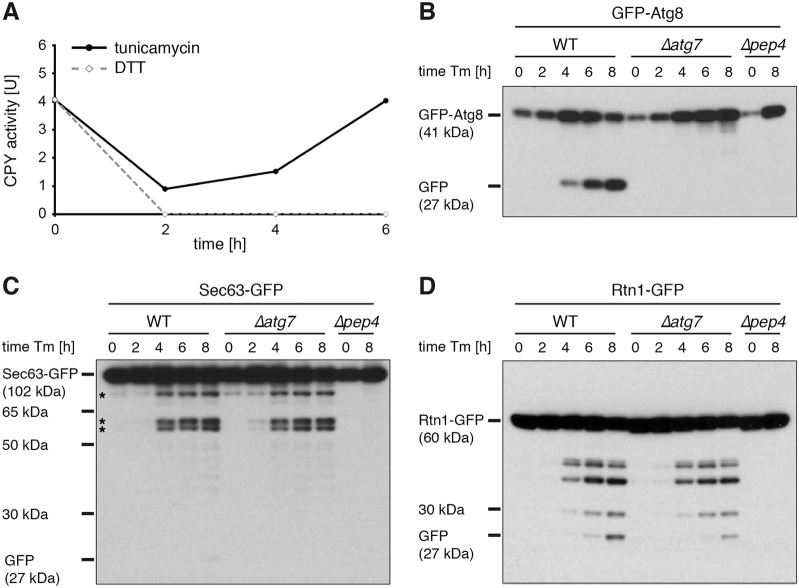
**ER stress triggers a composite autophagic response.** (A) CPY activity in enzyme units (U) in tunicamycin-treated and DTT-treated wild-type cells over time. (B) Western blot of GFP from wild-type (WT), *Δatg7* and PMSF-treated *Δpep4* cells expressing GFP–Atg8 and treated with tunicamycin for the times indicated. Tunicamycin induces the *ATG8* promoter so that the overall GFP signal increases with time. (C) As in B, but with strains expressing Sec63–GFP. Asterisks indicate Sec63–GFP fragments of ∼76, 63 and 59 kDa, whose generation required vacuolar proteolysis but not the core autophagy machinery. The GFP-containing cytosolic domain of Sec63–GFP is 76 kDa. (D) As in B but with strains expressing Rtn1–GFP. Tm, tunicamycin.

We first aimed to exploit the relative resistance of GFP to vacuolar proteolysis, which allows autophagy of GFP fusion proteins to be monitored by assessing the accumulation of GFP-containing cleavage products (Shintani and Klionsky, 2004). By measuring the activity of the vacuolar carboxypeptidase Y (CPY), we found that DTT blocked vacuolar proteolysis at the concentration we used, invalidating the cleavage assay (Fig. 4A). In contrast, tunicamycin inhibited CPY only transiently. Thus, tunicamycin is the ER stressor of choice for assays requiring vacuolar proteolysis. DTT, on the other hand, is ideal for morphological analyses because it is a powerful inducer of ER whorls but prevents their breakdown.

We then compared tunicamycin-triggered cleavage of the autophagosome marker GFP–Atg8 and the integral ER membrane protein Sec63–GFP. Tunicamycin induced GFP–Atg8 cleavage, consistent with the activation of macroautophagy by ER stress (Fig. 4B). As expected, cleavage was eliminated by removal of the core autophagy protein Atg7 or when vacuolar proteolysis was blocked by removal of the vacuolar aspartyl protease Pep4 combined with phenylmethylsulfonyl fluoride (PMSF) treatment to inhibit vacuolar serine proteases. Tunicamycin also elicited cleavage of Sec63–GFP, but the pattern of cleavage products was more complex (Fig. 4C). Besides a small amount of free GFP, three larger GFP-containing fragments were produced. These fragments were absent in a PMSF-treated *pep4* mutant and, hence, had arisen through vacuolar proteolysis. However, they persisted essentially unchanged in an *atg7* mutant. Sec63–GFP exposes its GFP-tagged C-terminus to the cytosol (Feldheim et al., 1992). The sizes of the Pep4-dependent but Atg7-independent cleavage products were the same or smaller than the cytosolic portion of Sec63–GFP. They could, therefore, only have been generated through proteolytic cuts in the cytosolic C-terminus of Sec63–GFP. These data indicate that the generation of the three larger fragments involves exposure of the cytosolic domain of Sec63–GFP to the lumen of the vacuole. We therefore infer that the larger fragments resulted from autophagy, albeit in an Atg7-independent manner.

As a confirmation, we analyzed Rtn1–GFP, which inserts into the ER membrane by means of hydrophobic hairpin structures and does not extend into the ER lumen (Voeltz et al., 2006). As for Sec63–GFP, tunicamycin induced the generation of GFP-containing cleavage products in wild-type and *atg7* mutant cells, but not in a PMSF-treated *pep4* mutant (Fig. 4D). Thus, tunicamycin causes cleavage of Rtn1–GFP in the vacuole through Atg7-independent autophagy.

We conclude that ER stress activates two autophagic mechanisms. One is reflected by the Atg7-dependent cleavage of GFP–Atg8 and the other by the Atg7-independent cleavage of the ER membrane proteins Sec63–GFP and Rtn1–GFP. In light of the observations made by electron microscopy, the simplest explanation is that these mechanisms correspond to non-selective macroautophagy and selective autophagy of ER whorls, respectively.

### Autophagy induced by ER stress selectively targets the ER

**Fig. 5.**
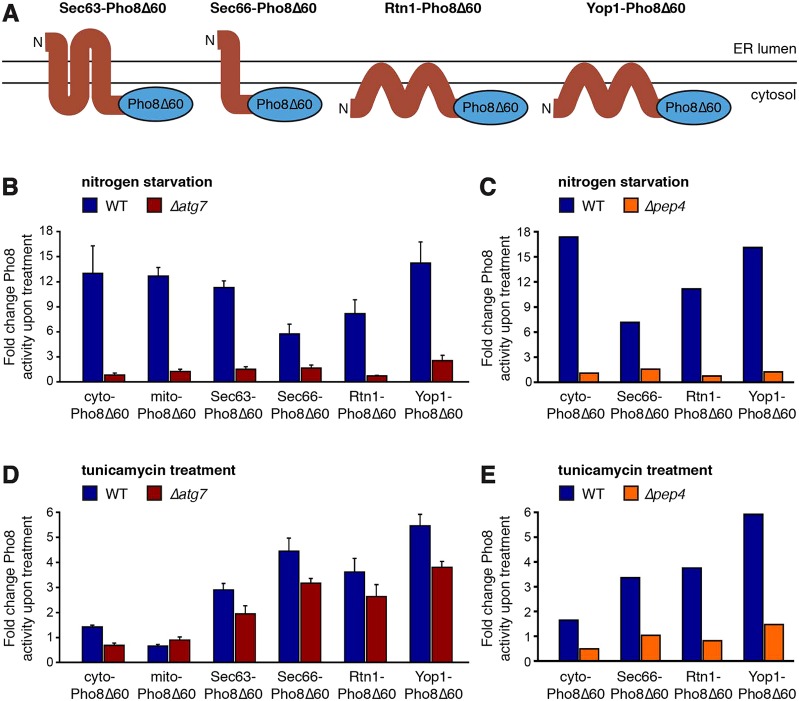
**ER stress-induced autophagy selectively targets the ER and does not require the core autophagy machinery.** (A) Schematics of the ER-localized reporters, all of which expose Pho8Δ60 to the cytosol. (B) Fold change of Pho8 activity of reporters for autophagy of cytosol (cyto-Pho8Δ60), mitochondria (mito-Pho8Δ60) and ER (Sec63–Pho8Δ60, Sec66–Pho8Δ60, Rtn1–Pho8Δ60 and Yop1–Pho8Δ60) upon nitrogen starvation in wild-type (WT) cells (blue bars) and *Δatg7* cells (dark red bars). Data are mean±s.e.m., *n* = 3. (C) Fold change of Pho8 activity of reporters for autophagy of cytosol (cyto-Pho8Δ60) and ER (Sec66–Pho8Δ60, Rtn1–Pho8Δ60 and Yop1–Pho8Δ60) upon nitrogen starvation in WT (blue bars) and PMSF-treated *Δpep4* cells (orange bars). (D) Fold change of Pho8 activity of the same reporters as in B upon tunicamycin treatment. Data are mean±s.e.m., *n* = 5. (E) Fold change of Pho8 activity of the same reporters as in C upon tunicamycin treatment.

As a second biochemical approach, we made use of Pho8Δ60 (Noda et al., 1995). Pho8 is a vacuolar phosphatase that is synthesized with an autoinhibitory propeptide and is therefore initially inactive. When Pho8 reaches the vacuole, its propeptide is cleaved off and its phosphatase activity is unmasked. Pho8Δ60 is a cytosolic version of Pho8 and has been used extensively as a marker for non-selective autophagy. In addition, Pho8Δ60 can be targeted to different organelles on whose autophagy it then reports. We generated strains in which Pho8Δ60 resided in the cytosol, in mitochondria or on the ER. Tethering of Pho8Δ60 to the ER was achieved by fusion to the cytosolic C-termini of the ER membrane proteins Sec63, Sec66, Rtn1 or Yop1 (Fig. 5A) (Feldheim et al., 1992; Feldheim et al., 1993; Voeltz et al., 2006). Induction of non-selective macroautophagy by nitrogen starvation activated all reporters in wild-type cells (Fig. 5B, blue bars). As expected, activation was blocked, that is, the fold change of reporter activities upon starvation was close to 1, in *atg7* mutants (Fig. 5B, red bars) and PMSF-treated *pep4* mutants (Fig. 5C). In contrast, tunicamycin elicited only a minor, ∼1.5-fold activation of cytosolic Pho8Δ60 in wild-type cells, did not induce the activity of mitochondrially targeted Pho8Δ60 and caused substantial activation only of the ER-localized reporters (Fig. 5D, blue bars). Activation was less pronounced compared to nitrogen starvation, either because starvation is a stronger autophagy inducer or because lower vacuolar proteolytic activity in tunicamycin-treated cells leads to inefficient removal of the Pho8 autoinhibitory propeptide. Deletion of *ATG7* abolished activation of cytosolic Pho8Δ60, suggesting that it had resulted from macroautophagy. Importantly, however, the fold activation of reporters for autophagy of ER was reduced by only ∼30% (Fig. 5D, dark red bars). The activation of ER-localized reporters was blocked in PMSF-treated *pep4* mutants, indicating that it took place in the vacuole and thus reflected autophagy (Fig. 5E). To rule out the possibility that this block was caused by effects of PMSF on non-vacuolar targets, we also tested tunicamycin-induced reporter activation in otherwise untreated *pep4* mutants. Activation was inhibited almost as strongly as with additional PMSF treatment, confirming reporter activation in the vacuole (supplementary material Fig. S4A).

These findings permit two conclusions. First, ER stress induces selective autophagy of </emph>ER. Second, autophagy of ER membrane proteins is largely insensitive to disruption of the core autophagy machinery and therefore occurs by a different mechanism than macroautophagy. We surmise that this mechanism is the selective autophagy of ER whorls observed by electron microscopy.

### ER-selective autophagy does not require known autophagic machinery

**Fig. 6.**
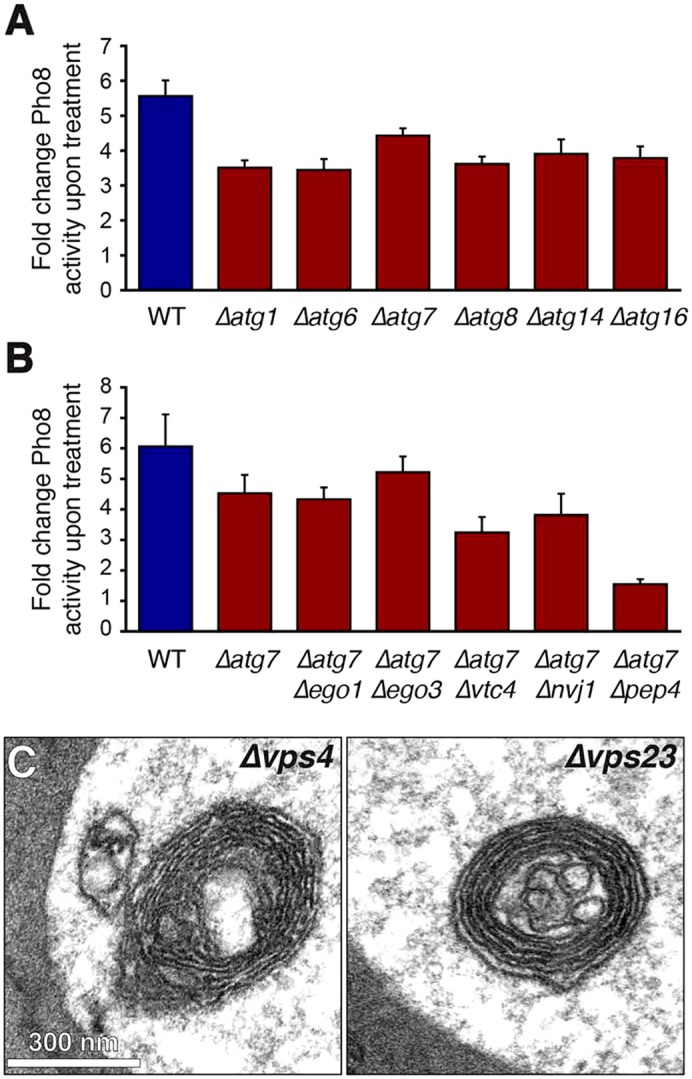
**Autophagy of ER does not require known autophagic machinery.** (A) Fold change of Pho8 activity of Yop1–Pho8Δ60 after tunicamycin treatment in wild-type (WT) cells (blue bar) and *Δatg1*, *Δatg6*, *Δatg7*, *Δatg8*, *Δatg14* and *Δatg16* cells (dark red bars). Data are mean±s.e.m., *n* = 3. (B) Fold change of Pho8 activity of Yop1–Pho8Δ60 after tunicamycin treatment in wild-type (WT) cells (blue bar) and *Δatg7* cells with or without additional deletion of *EGO1*, *EGO3*, *VTC4*, *NVJ1* or *PEP4* (dark red bars). Data are mean±s.e.m., *n* = 5. (C) Electron micrographs of ER whorls in the vacuoles of *Δvps4* and *Δvps23* cells treated with DTT for 3 h.

To further define selective autophagy of ER, we systematically tested its requirement for known autophagic machinery. We first wanted to corroborate that it does not require the core autophagy machinery. To this end, we measured tunicamycin-induced Yop1–Pho8Δ60 activation in mutants lacking Atg1, Atg6, Atg7, Atg8, Atg14 or Atg16. Activation of Yop1–Pho8Δ60 showed a reduction of ∼30% in all of these mutants (Fig. 6A). This finding confirms that the core autophagy machinery is not essential for autophagy of ER.

Next, we tested whether autophagy of ER required genes specifically implicated in microautophagy. To focus on autophagy that is independent of the known core autophagy machinery, we first deleted *ATG7*. We then additionally deleted *EGO1*, *EGO3*, *VTC4* or *NVJ1*, which are essential components of the EGO complex, the VTC complex and the nucleus–vacuole junction, respectively, and have roles in non-selective microautophagy and microautophagy of the nucleus (Roberts et al., 2003; Dubouloz et al., 2005; Uttenweiler et al., 2007). None of the double mutants showed a significant defect in tunicamycin-induced activation of Yop1–Pho8Δ60 compared to the *atg7* single mutant (*P*>0.1), whereas an *atg7 pep4* double mutant did (*P*<0.005; Fig. 6B). We confirmed by electron microscopy that ER whorls still formed and were delivered to the vacuole in *ego1*, *ego3*, *vtc4*, *nvj1* and *pep4* mutants (data not shown). Thus, autophagy of the ER does not require the EGO complex, the VTC complex or the nucleus–vacuole junction.

The endosomal sorting complexes required for transport (ESCRT) machinery mediates inward budding of late endosomes and has been linked to microautophagy (Sahu et al., 2011). Deletion of the ESCRT genes *VPS4*, *VPS23* or *VPS24* abolished activation of Yop1–Pho8Δ60 (supplementary material Fig. S4B). However, the ESCRT machinery is indirectly needed for transport of proteases to the vacuole (Bowers and Stevens, 2005), as reflected by strongly diminished CPY activity in *vps4*, *vps23* and *vps24* mutants (supplementary material Fig. S4C). Hence, ESCRT mutants are likely unable to remove the inhibitory propeptide from Pho8, rendering the Yop1–Pho8Δ60 reporter uninformative. We therefore turned to electron microscopy to evaluate autophagy of ER whorls in mutants lacking ESCRT components. Both *vps4* and *vps23* mutants still showed ER whorls in the vacuole during ER stress (Fig. 6C). These qualitative morphological data do not exclude a role for the ESCRT machinery in the autophagy of ER whorls, but it is clearly not essential.

### ER-selective autophagy degrades excess ER

**Fig. 7.**
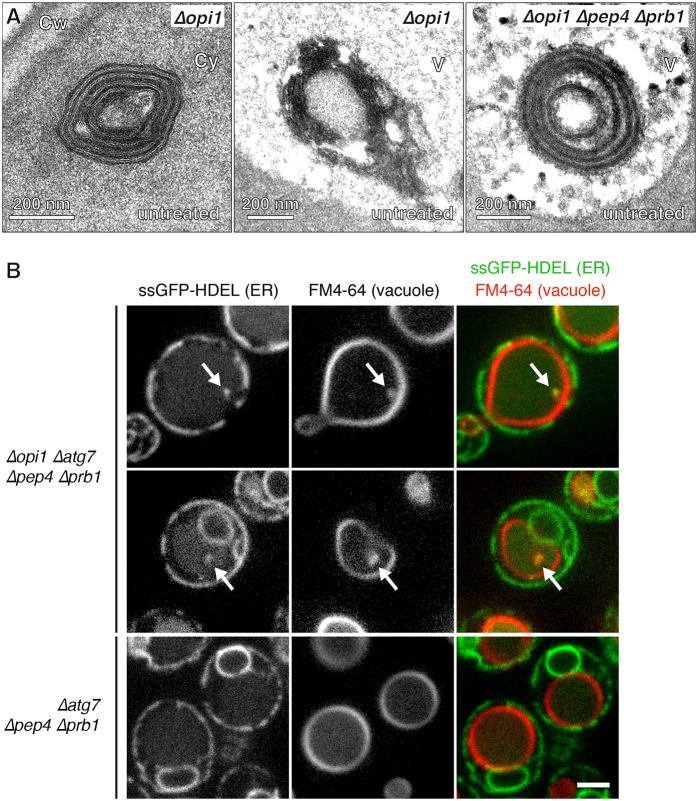
**Degradation of excess ER by selective autophagy.** (A) Electron micrographs of untreated *Δopi1* and *Δopi1 Δpep4 Δprb1* cells. Cw, cell wall; Cy, cytoplasm; V, vacuole. (B) Confocal images of *Δopi1 Δatg7 Δpep4 Δprb1* and *Δatg7 Δpep4 Δprb1* cells expressing the ER marker ssGFP-HDEL. Cells were stained with the vacuolar membrane dye FM4-64 but otherwise untreated. Arrows point to ER inside the vacuole where it colocalizes with vacuolar membrane. Scale bar: 2 µm.

Finally, to begin to address the physiological functions of ER-selective autophagy, we tested whether autophagy of ER whorls required accumulation of misfolded proteins in the ER or whether expansion of the ER membrane, which is an integral part of the normal response to ER stress, is sufficient. Experimentally, membrane expansion without ER stress can be achieved by activating lipid biosynthesis through removal of the transcriptional repressor Opi1 (Schuck et al., 2009). Exponentially growing, untreated *opi1* mutants exhibited ER whorls in the cytoplasm, showing that membrane expansion can trigger whorl formation (Fig. 7A, left). In addition, partially disintegrated ER whorls were observed in the vacuole, suggesting that they are being degraded there (Fig. 7A, middle). Such degradation intermediates had not been observed after DTT or tunicamycin treatment, likely because these reagents interfere with vacuolar proteolysis (Fig. 4A). To test this explanation, we analyzed *opi1 pep4 prb1* triple mutants. Pep4 and Prb1 are needed for the proteolytic activation of many vacuolar hydrolases, likely including the lipases responsible for disassembling ER whorls. Indeed, intact ER whorls were present in the vacuole of untreated *opi1 pep4 prb1* mutants (Fig. 7A, right). In contrast, no whorls could be detected in untreated *pep4 prb1* mutants, excluding the possibility that blocking vacuolar degradation by itself causes accumulation of ER whorls (data not shown). Therefore, autophagy of ER whorls can be elicited by ER membrane expansion even in the absence of misfolded proteins. This finding suggests that organelle-selective autophagy limits ER expansion and controls ER size.

To corroborate these results by light microscopy, we visualized the ER by expression of ssGFP-HDEL, which contains an ER-targeting signal sequence as well as an ER retention signal, and stained the vacuolar membrane with the lipophilic dye FM4-64 (Vida and Emr, 1995). When these markers were analyzed in untreated *opi1 atg7 pep4 prb1* mutant cells, ssGFP-HDEL-positive dots were found inside the vacuole, where they colocalized with FM4-64 (Fig. 7B, top two panels). No such dots were observed in *atg7 pep4 prb1* cells with an intact *OPI1* gene (Fig. 7B, bottom panels). These data show that ER is constitutively transported into the vacuole in *opi1* mutants and that this process does not require the core autophagy machinery. Furthermore, colocalization of ER with vacuolar membrane inside the vacuole indicates an uptake mechanism that involves inward budding of the vacuolar membrane, in agreement with the electron microscopic observations.

## DISCUSSION

**Fig. 8.**
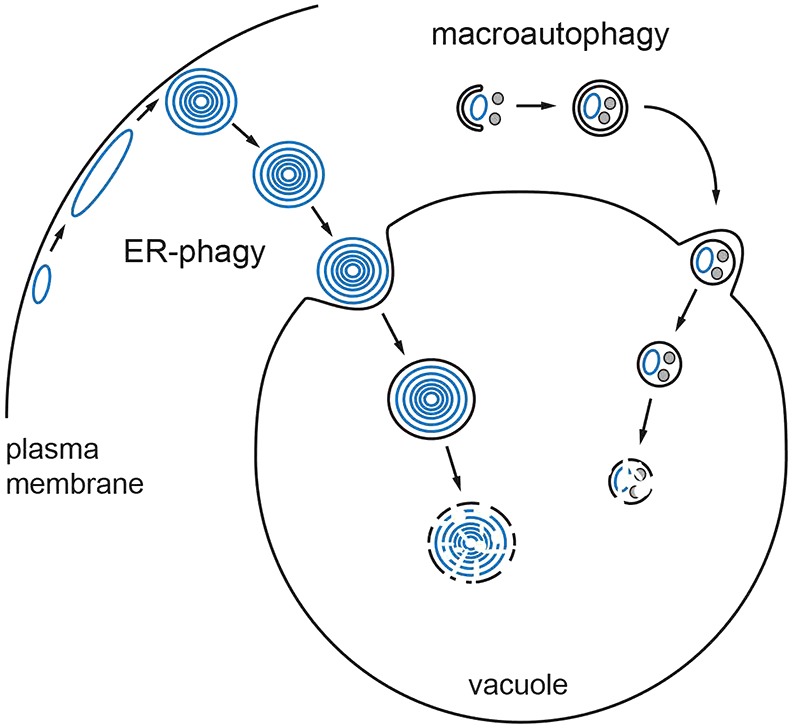
**Model of the autophagic response to ER stress.** ER stress induces expansion of the peripheral ER (blue) and formation of ER whorls, which are selectively taken up into the vacuole by ER-phagy. Concomitantly, macroautophagy is activated. Forming autophagosomes (crescent-shaped membrane sac) engulf pieces of the ER (blue) as well as other cytoplasmic constituents (gray). ER-phagy and macroautophagy might act independently and in parallel, as shown in this model, but could also be linked.

The present study builds on earlier work describing the formation of ER whorls (Bernales et al., 2006). Here, we have shown that stress-induced ER whorls are taken up into the yeast lysosome by a new type of autophagy, which we call ER-phagy. It is distinguished from all previously known types of autophagy by a unique combination of features. ER-phagy is: (1) an autophagic process topologically equivalent to microautophagy, (2) organelle-selective, yet (3) independent of the core autophagy machinery, and (4) independent of other components linked to microautophagy, including the EGO complex, the VTC complex, the nucleus–vacuole junction and the ESCRT machinery. Hence, ER-phagy occurs by a distinctive molecular mechanism that remains to be elucidated. This mechanism must bring about a series of fascinating cellular events, including the generation of spherical membrane whorls from cisternal ER, the recognition of these whorls as structures to be degraded, their severance from the remainder of the ER, their selective engulfment by the vacuolar membrane and finally their uptake into the vacuole by inward budding (Fig. 8).

This study also delineates ER-phagy and macroautophagy as two components of the composite autophagic response to ER stress. These components were previously conflated when ER whorls were proposed to be autophagosomes and ER-phagy was classified as a type of macroautophagy (Bernales et al., 2006; Bernales et al., 2007). This confusion arose because ER whorls were not detected in an *atg8* mutant (Bernales et al., 2006). We show here that ER whorls still form in the absence of Atg1, Atg7, Atg8 or Atg16, demonstrating that ER whorls are not autophagosomes. Furthermore, we show that ER whorls are delivered into the vacuole during ER stress, in contrast to the earlier suggestion that delivery takes place only after the stress has abated (Bernales et al., 2006). The reasons for these discrepancies between this and the earlier work remain unclear. Further resolution will have to await the molecular definition of ER-phagy by identification of the machinery that obligatorily or facilitatively mediates the observed morphological events.

Why both ER-phagy and macroautophagy are activated by ER stress is unclear. The two processes could operate independently, with ER-phagy acting through ER whorls and macroautophagy targeting the ER by means of autophagosomes (Fig. 8). Alternatively, macroautophagy could indirectly contribute to ER-phagy. It has been suggested that starvation-induced macroautophagy in yeast entails microautophagy to balance the expansion of the vacuolar membrane that results from autophagosome fusion with the vacuole (Müller et al., 2000). Conversely, ER-phagy consumes vacuolar membrane every time an ER whorl is sequestered, and this loss might be compensated for by macroautophagy. Nevertheless, deletion of core autophagy genes causes only a mild reduction of ER-phagy as measured biochemically, indicating that macroautophagy has at most a minor role. Accordingly, we hypothesize that the physiological benefit of the autophagic response to ER stress is mainly provided by ER-phagy rather than macroautophagy. Consistent with this notion, deletion of core autophagy genes does not impair cell proliferation in the presence of tunicamycin. These data conflict with previous results showing tunicamycin-dependent growth defects of macroautophagy mutants (Bernales et al., 2006). However, we have found since that these earlier observations can be attributed, at least in part, to the fact that the strains used were not completely isogenic (supplementary material Fig. S5).

It has been postulated previously that ER-phagy counterbalances ER expansion during ER stress (Bernales et al., 2006). Here, we have provided evidence that ER-phagy indeed functions to control ER size. Unchecked lipid biosynthesis in the absence of Opi1 results in the generation of superfluous ER membrane (Schuck et al., 2009). The concomitant activation of ER-phagy implies that cells are able to detect this overabundance and act to eliminate it by organelle-selective autophagy. Thus, ER-phagy functions as a counterpart to the unfolded protein response, which enables cells to sense an unmet demand for ER function and expand their ER accordingly (Walter and Ron, 2011). Together, the unfolded protein response and ER-phagy might constitute a regulatory circuit that homeostatically adjusts ER abundance to current demand.

Whether ER-phagy also exists in higher eukaryotes is an open question. However, ER whorls have been observed in mammalian cells a number of times and have even been linked to a poorly characterized autophagic response (Bolender and Weibel, 1973; Borgese et al., 2006; Lingwood et al., 2009). Furthermore, the situation in *opi1* mutant yeast is conceptually similar to that in hepatocytes that are first treated with inducers of the cytochrome P450 detoxification system, to bring about ER expansion, and then are released from drug treatment. Once the stimulus for ER expansion is removed, a portion of the ER becomes redundant and is degraded through selective autophagy of whorled ER membranes (Bolender and Weibel, 1973). Whether these similarities to yeast extend to the underlying autophagic mechanisms remains to be established.

## MATERIALS AND METHODS

### Plasmids

All plasmids used in this study are listed in supplementary material Table S1. To generate pFA6a-Pho8Δ60-kanMX6 for gene tagging with Pho8Δ60, Pho8(60–567) was amplified from yeast genomic DNA and cloned into the PacI/AscI sites of pFA6a-GFP(S65T)-kanMX6 (Longtine et al., 1998), replacing GFP(S65T). To generate p306-ADH-CoxIV-Pho8Δ60 for expression of mitochondrial Pho8Δ60, ADH-CoxIV-Pho8Δ60 from pCC4 (Campbell and Thorsness, 1998) was subcloned into the KpnI/BamHI sites of pRS306 (Sikorski and Hieter, 1989). To generate YIplac-ssGFPHDEL-NatMX, GFP was amplified from pFA6a-GFP(S65T)-His3MX6 (Longtine et al., 1998) and cloned into the SpeI/XbaI sites of YIplac-ssDsRedHDEL-NatMX (Madrid et al., 2006), replacing DsRed.

### Yeast strains

All strains were derived from wild-type W303 mating type **a** and are listed in supplementary material Table S2. Chromosomal integrations and replacements were introduced by homologous recombination using PCR products (Longtine et al., 1998; Janke et al., 2004) or by means of the integrative plasmids described above. Gene tagging or deletion was confirmed by colony PCR. Deletion of core autophagy genes was additionally confirmed by loss of autophagic body accumulation upon treatment with 0.2 µg/ml rapamycin (Sigma) and 1 mM PMSF (Sigma) for 6 h as judged by light microscopy.

### Electron microscopy

Cultures grown to early log phase [optical density at 600 nm (OD_600_) = 0.2–0.3] at 30°C in YPD medium (yeast extract, peptone, 2% dextrose) were left untreated for 1.5 h or were treated with 8 mM DTT (Roche) or 1 µg/ml tunicamycin (EMD) for up to 4 h. Cells were fixed, stained with KMnO_4_ and embedded as described previously (Bernales et al., 2006), except that epon resin was used. Thin sections (50 or 70 nm) were cut, stained with 2% aqueous uranyl acetate and Reynold's lead citrate, and viewed with an FEI Tecnai 12 transmission electron microscope.

### Western blotting

Cultures were grown to early log-phase in YPD or in SC medium (yeast nitrogen base, amino acids, 2% dextrose) lacking uracil. Cells were treated with 1 µg/ml tunicamycin. 1 mM PMSF was added to all *Δpep4* strains and refreshed after 4 h in order to block vacuolar proteolysis completely. After the times indicated, cells were pelleted, proteins were extracted with urea and SDS, and protein concentrations were measured with the bicinchoninic acid protein assay kit (Thermo Fisher Scientific). Equal amounts of total protein were resolved on Bis-Tris gels (Invitrogen) and transferred onto nitrocellulose membranes. Equal loading and even transfer were confirmed by Ponceau S staining. GFP was detected with mouse anti-GFP antibodies 7.1/13.1 (Roche).

### CPY assay

To determine CPY activity, mid-log-phase cultures (OD_600_ = 0.5) in YPD were left untreated for 2 h, or treated with 1 µg/ml tunicamycin or 8 mM DTT for up to 6 h. All cultures were OD_600_ = 1.0–1.5 when they were harvested, which was important because CPY activity increases with culture density (Jones, 2002). The CPY assay was based on that described previously (Jones, 2002). Cells (equivalent to 1 ml culture of OD_600_ = 30) were pelleted, washed once with water and resuspended in 200 µl ice-cold Tris buffer (100 mM Tris-HCl pH 7.6). Cells were lysed by bead beating, debris was pelleted and protein concentrations of the resulting lysates were determined as above. Reactions consisting of 50 µl lysate, 150 µl Tris buffer and 50 µl 5 mM N-benzoyl-L-tyrosine p-nitroanilide (Sigma) in dimethylformamide were incubated at 37°C for 60 min, then stopped with 230 µl ice-cold 6 M urea in Tris buffer and 1 µl 1 M PMSF in DMSO. After 15 min on ice, 20 µl 25% SDS was added and samples were cleared by incubation at 65°C for 2 min. Absorption at 405 nm was used to measure p-nitroaniline concentrations. One unit CPY activity was defined as 10 pmol p-nitroaniline produced per min and mg of protein.

### Pho8 assay

To determine Pho8 activity, mid-log-phase cultures in YPD medium were left untreated for 3 h, treated with 1 µg/ml tunicamycin for 9 h or were pelleted, resuspended in the same volume of nitrogen-free SC-N starvation medium (yeast nitrogen base without ammonium sulfate, 2% dextrose) and grown for 9 h. *Pep4* mutant strains were additionally treated with 1 mM PMSF where indicated. The Pho8 assay was carried out as described previously (Noda and Klionsky, 2008) using p-nitrophenyl phosphate (Sigma) as substrate. One unit alkaline phosphatase activity was defined as 1 nmol p-nitrophenol produced per min and mg of protein. To obtain values for fold induction, the activities measured in treated and untreated reporter strains were first corrected by subtracting background activities measured in identically treated control strains not expressing a reporter. Corrected Pho8 activities of treated samples were then divided by those of the corresponding untreated samples. Where indicated, statistical significance was determined by Student's *t*-test.

### Light microscopy

Cultures were grown to early log phase in SC medium and labeled with 2 µg/ml FM4-64 (Life Technologies) for 1 h at 30°C. Cells were washed twice with SC medium, grown for another 1 h, transferred onto a glass slide and imaged live at room temperature. Images were acquired with a Nikon Ti-E inverted microscope equipped with a Yokagawa CSU-X1 spinning disk confocal scanner unit, two Andor iXon EM-CCD cameras as part of a dual camera set-up and a Nikon CFI APO TIRF 100×/1.49 NA oil objective lens. The microscope was controlled by MicroManager software (Edelstein et al., 2010). Both GFP and FM4-64 were excited at 488 nm with an Agilent MLC400B monolithic laser combiner and imaged simultaneously using 525/50 and 700/75 bandpass emission filters, respectively. The brightness and contrast of the resulting images was adjusted using Adobe Photoshop.

## Supplementary Material

Supplementary Material
