# Supplementary material for: ER-phagy mediates selective degradation of endoplasmic reticulum independently of the core autophagy machinery
Source: J Cell Sci. 2014 Sep 15;127(18):4078–88. doi: 10.1242/jcs.154716 (PMC4163648; doi:10.1242/jcs.154716)
Supplement: Supplementary Material [file supp_127_18_4078__index.html]

ER-phagy mediates selective degradation of endoplasmic reticulum independently of the core autophagy machinery — Supplementary Material 

# ER-phagy mediates selective degradation of endoplasmic reticulum independently of the core autophagy machinery

## JCS154716 Supplementary Material

**Files in this Data Supplement:**

- **Supplementary Material**
