# Supplementary material for: ER-phagy mediates selective degradation of endoplasmic reticulum independently of the core autophagy machinery
Source: J Cell Sci. 2014 Sep 15;127(18):4078–88. doi: 10.1242/jcs.154716 (PMC4163648; doi:10.1242/jcs.154716)
Supplement: Supplementary Material [file supp_127.18.4078_JCS154716.pdf]

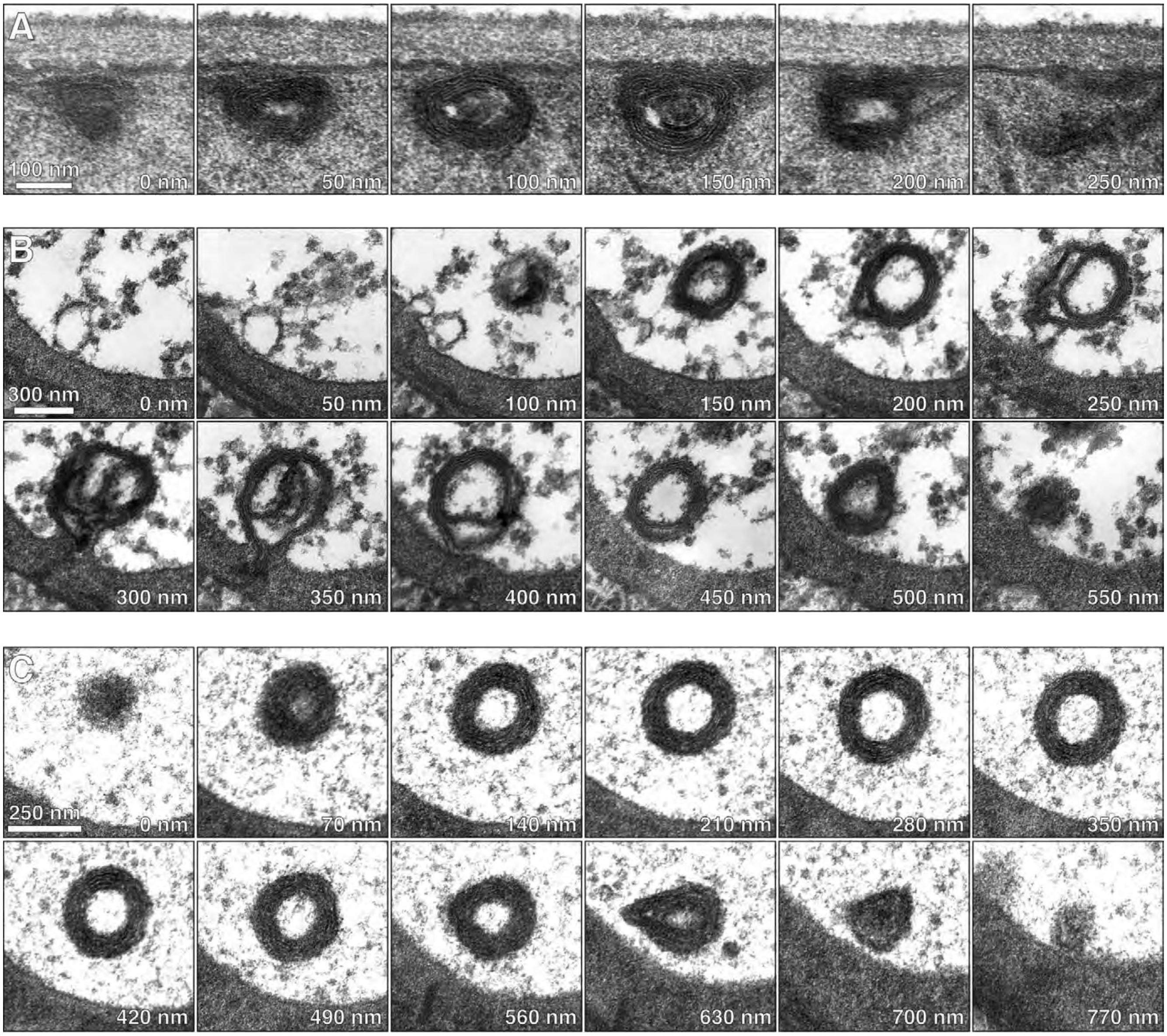

Fig. S1. Stages of autophagy of ER whorls. **(A)** Electron micrographs of serial 50 nm thin sections showing an ER whorl underneath the plasma membrane. **(B)** Electron micrographs of serial 50 nm thin sections showing an ER whorl in the vacuole that is still connected to the cortical ER through a narrow neck (visible in sections 300 nm and 350 nm). The micrograph of section 350 nm is a lower magnification image of Figure 1F. **(C)** Electron micrographs of serial 70 nm thin sections showing an ER whorl in the vacuole that is disconnected from the cytosol.

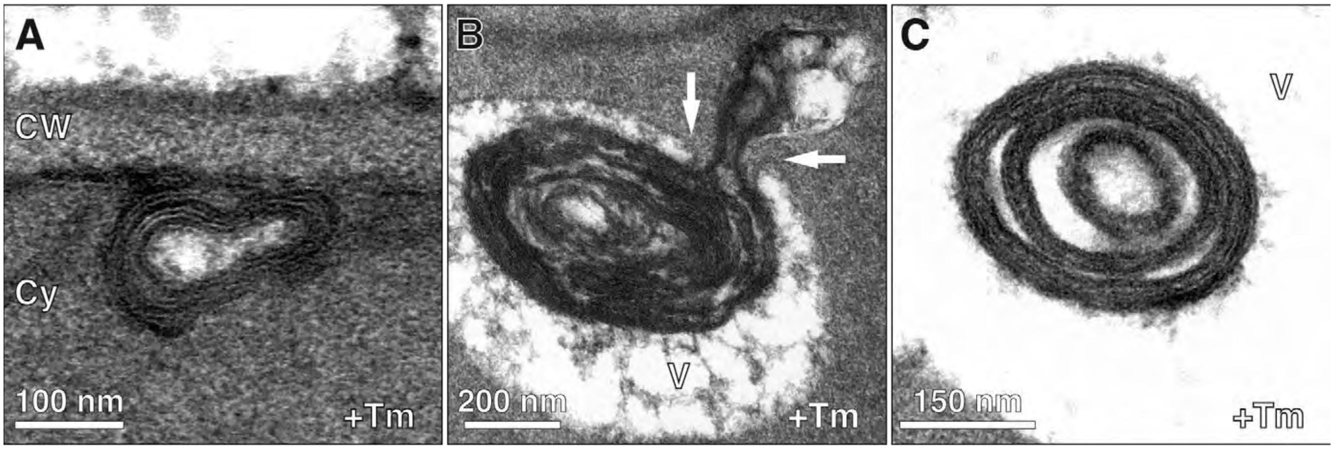

Fig. S2. Autophagy of ER whorls upon tunicamycin treatment. Electron micrographs of wild-type yeast treated with tunicamycin for 4 h, illustrating **(A)** ER whorl formation at the cell cortex, **(B)** uptake into the vacuole by invagination of the vacuolar membrane (white arrows), and **(C)** completed import into the vacuole. CW, cell wall; Cy, cytoplasm; Tm, tunicamycin; V, vacuole.

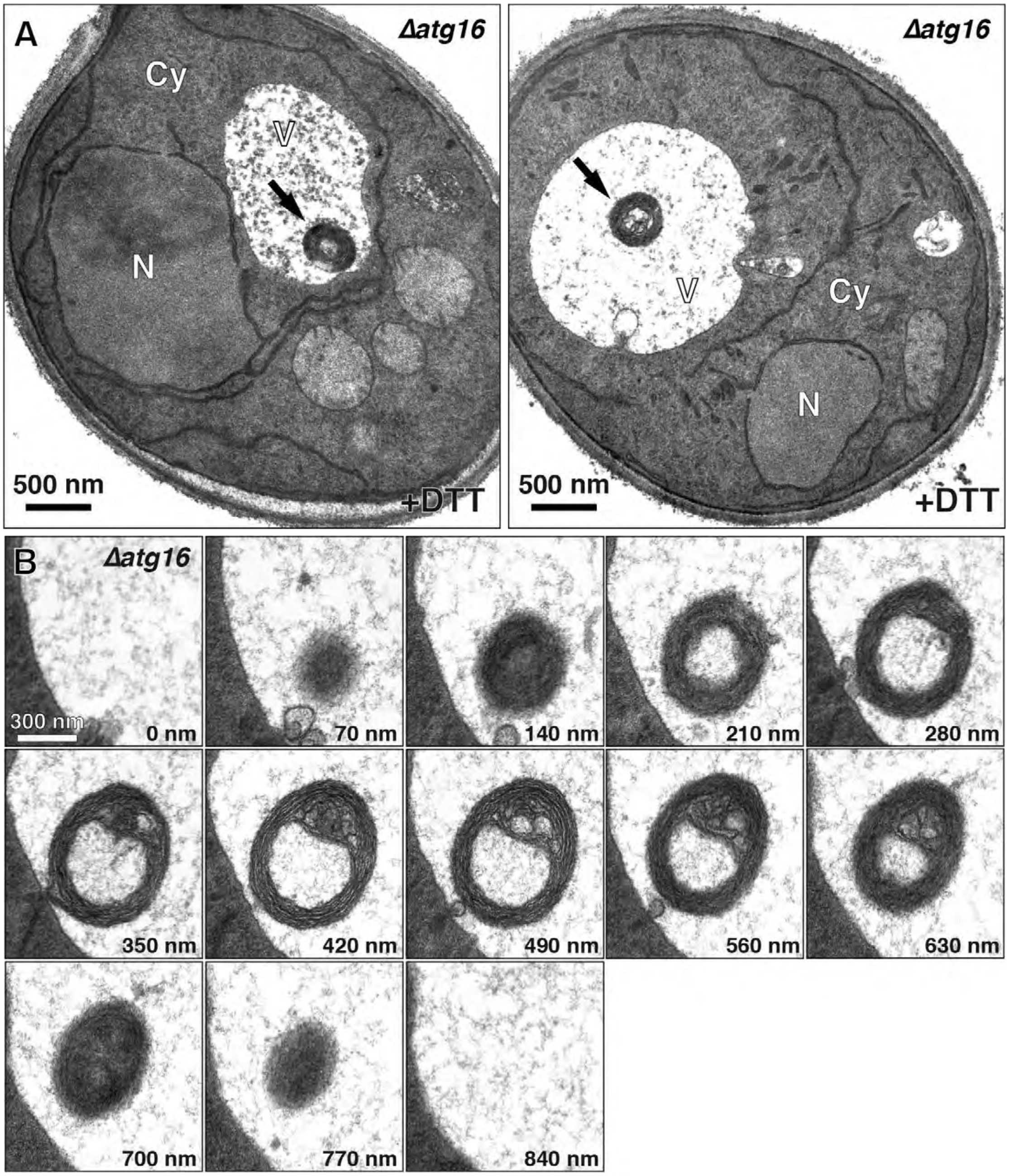

Fig. S3. Autophagy of ER whorls does not require key *ATG* genes. **(A, B)** Low magnification electron micrographs of ER whorls (black arrows) in the vacuoles of *Δatg16* cells treated with DTT for 3 h. **(C)** Electron micrographs of serial 70 nm thin sections showing an ER whorl that has been taken up into the vacuole of a *Δatg16* cell treated with DTT for 3 h. Cy, cytoplasm; N, nucleus; V, vacuole.

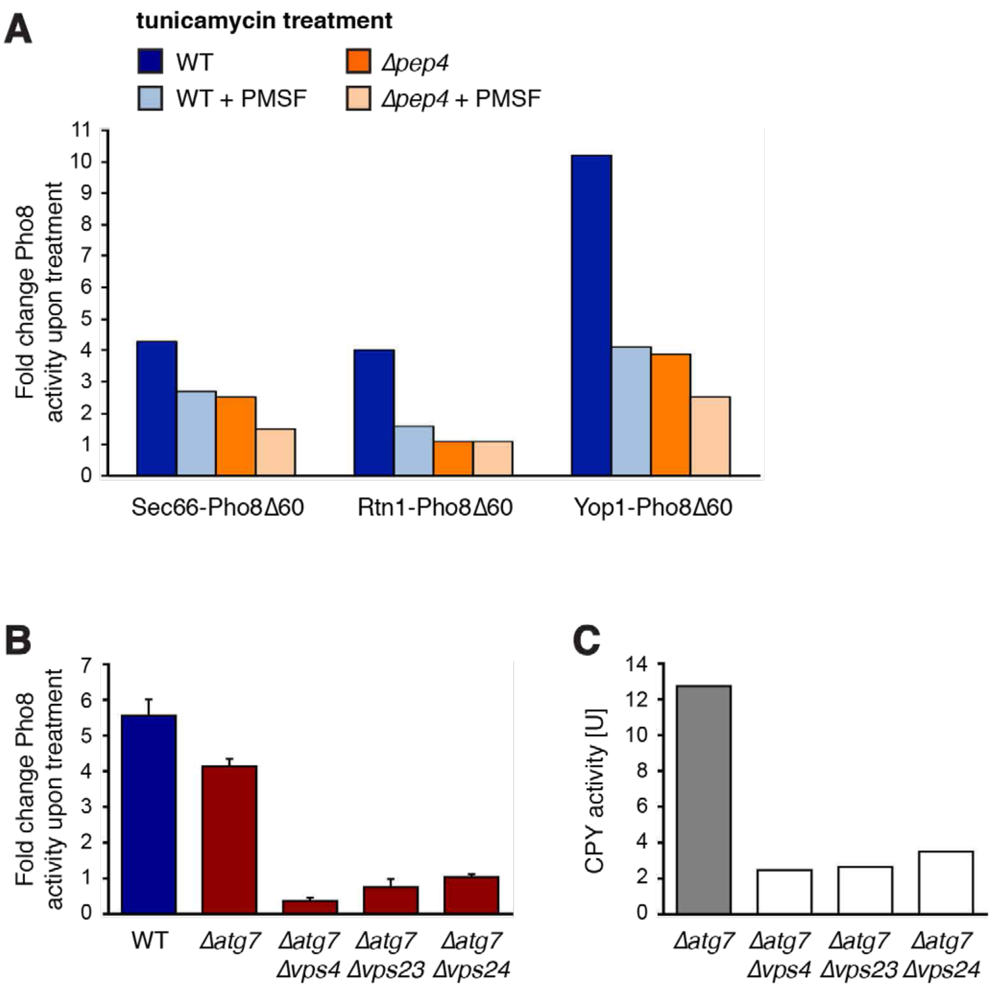

Fig. S4. Impairment of ER-localized Pho8Δ60 reporter activation and CPY activity in *pep4* and ESCRT mutants. **(A)** Fold change of Pho8 activity of Sec66-Pho8Δ60, Rtn1-Pho8Δ60, Yop1-Pho8Δ60 upon tunicamycin treatment in wild-type (WT) cells (blue bars) and *Δpep4* cells (orange bars) without and with additional PMSF treatment (dark and light bars, respectively). **(B)** Fold change of Pho8 activity of Yop1-Pho8Δ60 after tunicamycin treatment in WT cells (blue bar) and *Δatg7* cells with or without additional deletion of *VPS4*, *VPS23* or *VPS24* (red bars). Data are mean ± SEM, n = 3. **(C)** CPY activity in enzyme units U in *Δatg7* cells (grey bar) and cells with additional deletion of *VPS4*, *VPS23* or *VPS24* (white bars).

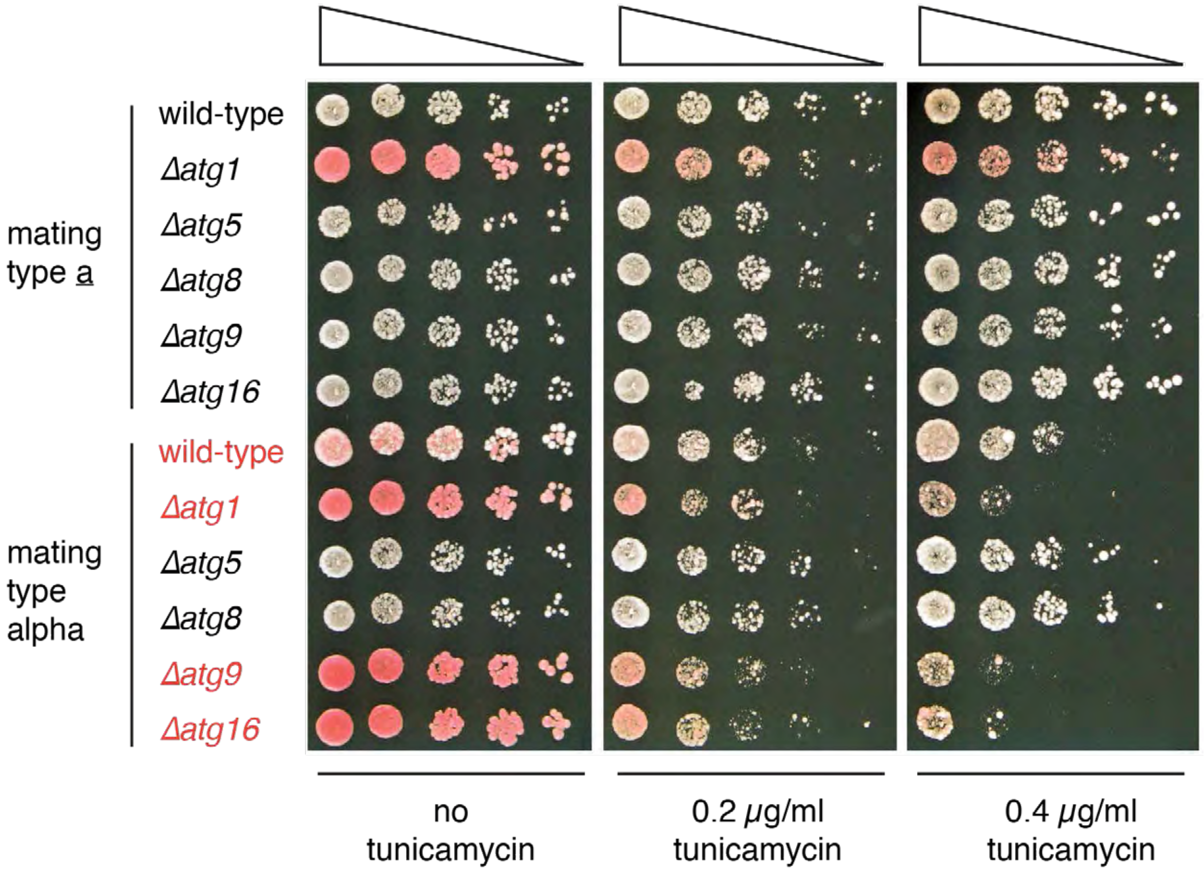

Fig. S5. Deletion of autophagy genes does not impair growth in the presence of tunicamycin. Exponentially growing wild-type and *atg* mutant cells were serially diluted in 4-fold dilution steps, spotted onto YPD plates containing tunicamycin at the concentrations indicated and grown for 2 days (no and 0.2 µg/ml tunicamycin) or 4 days (0.4 µg/ml tunicamycin) at 30ºC. Strains marked in red were derived previously (Bernales *et al.*, 2006). All mating type a strains grew equally well under all conditions. The mating type alpha wild-type strain grew more slowly on tunicamycin plates than the mating type a wild-type strain. The previously used mating type alpha *Δatg1*, *Δatg9* and *Δatg16* mutants grew worse at high tunicamycin concentrations than the corresponding wild-type, as reported by Bernales et al., 2006. However, the newly derived mating type alpha *Δatg5* and *Δatg8* mutants grew better on tunicamycin than both the previously used *atg* mutants and the mating type alpha wild-type. Qualitatively similar results were obtained with higher tunicamycin concentrations, growth at 37ºC or a combination of both. These observations indicate that the growth differences observed by Bernales *et al.*, 2006 are unrelated to deletions of core autophagy genes, all of which are functionally equivalent. A possible explanation is provided by the observations that the mating type alpha wild-type is a mixture of white and red colonies, that the previously used *atg* mutants are uniformly red whereas the newly derived *atg* mutants are uniformly white, and that the growth phenotypes segregate with the red colour. The red colour arises through mutation of the *ADE2* gene, which leads to accumulation of toxic red intermediates of adenin biosynthesis that impede growth. The white colonies have acquired suppressor mutations that prevent accumulation of the toxic red pigments. Hence, the mating type alpha wild-type and *atg* mutant strains used previously are not isogenic and their different growth behaviours on tunicamycin-containing plates is likely caused by mutations in the adenin biosynthesis pathway.

Table S1. Plasmids used in this study.

Plasmid	Source
pRS316-GFPAtg8	Suzuki et al., 2001
pFA6a-GFP(S65T)-kanMX6	Longtine et al., 1998
pFA6a-Pho8Δ60-kanMX6	this study
pCC4	Campbell and Thorsness, 1998
pRS306	Sikorski and Hieter, 1989
p306-ADH-CoxIV-Pho8Δ60	this study
pFA6a-GFP(S65T)-His3MX6	Longtine et al., 1998
YIplac-ssDsRedHDEL-NatMX	Madrid et al., 2006
YIplac-ssGFPHDEL-NatMX	this study

Publication: Journal of Cell Science (US spelling)
Type: Research Article; Open Access: F; Volume: ; Issue:

Table S2. Yeast strains used in this study.

Strain	Relevant genotype	Source
W303	*leu2-3,112 trp1-1 ura3-1 his3-11,15 mat a*	Walter lab
SSY005	*atg1Δ::kan*	this study
SSY013	*atg7Δ::kan*	this study
SSY015	*atg8Δ::HIS3*	this study
SSY023	*atg16Δ::kan*	this study
SSY321	*ego1Δ::HIS3*	this study
SSY322	*ego3Δ::HIS3*	this study
SSY323	*vtc4Δ::HIS3*	this study
SSY324	*nvj1Δ::HIS3*	this study
PWY005	*pep4Δ::TRP1*	this study
SSY003	*vps4Δ::HIS3*	this study
SSY058	*vps23Δ::HIS3*	this study
SSY071	pRS316-GFPATG8	this study
SSY072	*atg7Δ::kan* pRS316-GFPATG8	this study
SSY073	*pep4Δ::TRP1* pRS316-GFPATG8	this study
SSY139	*SEC63-GFP::HIS3 VPH1-cherry::TRP1*	Schuck et 2009
SSY243	*SEC63-GFP::HIS3 VPH1-cherry::TRP1 atg7Δ::kan*	this study
SSY244	*SEC63-GFP::HIS3 VPH1-cherry::kan pep4Δ::TRP1*	this study
SSY228	*RTN1-GFP::TRP1*	this study
SSY273	*RTN1-GFP::TRP1 atg7Δ::kan*	this study
SSY274	*RTN1-GFP::TRP1 pep4Δ::kan*	this study
SSY605	*pho8Δ::HIS3 pho13Δ::TRP*	this study
SSY609	*pho8Δ::HIS3 pho13Δ::TRP atg7Δ::nat*	this study
SSY610	*pho8Δ::HIS3 pho13Δ::TRP pep4Δ::nat*	this study
SSY614	*pho13Δ::HIS3* P_{GPD}*-pho8Δ60::kan*	this study

Strain	Relevant genotype	Source
SSY615	*pho13Δ::HIS3 P_{GPD}-pho8Δ60::kan atg7Δ::nat*	this study
SSY616	*pho13Δ::HIS3 P_{GPD}-pho8Δ60::kan pep4Δ::nat*	this study
SSY627	*pho8Δ::HIS3 pho13Δ::TRP URA3::P_{ADH}-COXIV-pho8Δ60*	this study
SSY628	*pho8Δ::HIS3 pho13Δ::TRP atg7Δ::nat URA3::P_{ADH}-COXIV-pho8Δ60*	this study
SSY613	*pho8Δ::HIS3 pho13Δ::TRP SEC63-pho8Δ60::kan*	this study
SSY617	*pho8Δ::HIS3 pho13Δ::TRP SEC63-pho8Δ60::kan atg7Δ::nat*	this study
SSY630	*pho8Δ::HIS3 pho13Δ::TRP SEC66-pho8Δ60::kan*	this study
SSY641	*pho8Δ::HIS3 pho13Δ::TRP SEC66-pho8Δ60::kan atg7Δ::nat*	this study
SSY642	*pho8Δ::HIS3 pho13Δ::TRP SEC66-pho8Δ60::kan pep4Δ::nat*	this study
SSY612	*pho8Δ::HIS3 pho13Δ::TRP RTN1-pho8Δ60::kan*	this study
SSY634	*pho8Δ::HIS3 pho13Δ::TRP RTN1-pho8Δ60::kan atg7Δ::nat*	this study
SSY635	*pho8Δ::HIS3 pho13Δ::TRP RTN1-pho8Δ60::kan pep4Δ::nat*	this study
SSY611	*pho8Δ::HIS3 pho13Δ::TRP YOP1-pho8Δ60::kan*	this study
SSY621	*pho8Δ::HIS3 pho13Δ::TRP YOP1-pho8Δ60::kan atg7Δ::nat*	this study
SSY622	*pho8Δ::HIS3 pho13Δ::TRP YOP1-pho8Δ60::kan pep4Δ::nat*	this study
SSY651	*pho8Δ::HIS3 pho13Δ::TRP YOP1-pho8Δ60::kan atg1Δ::nat*	this study
SSY652	*pho8Δ::HIS3 pho13Δ::TRP YOP1-pho8Δ60::kan atg6Δ::nat*	this study
SSY653	*pho8Δ::HIS3 pho13Δ::TRP YOP1-pho8Δ60::kan atg8Δ::nat*	this study
SSY654	*pho8Δ::HIS3 pho13Δ::TRP YOP1-pho8Δ60::kan atg14Δ::nat*	this study
SSY656	*pho8Δ::HIS3 pho13Δ::TRP YOP1-pho8Δ60::kan atg16Δ::nat*	this study
SSY657	*pho8Δ::HIS3 pho13Δ::TRP YOP1-pho8Δ60::kan atg7Δ::LEU2*	this study
SSY658	*pho8Δ::HIS3 pho13Δ::TRP YOP1-pho8Δ60::kan atg7Δ::LEU2 ego1Δ::nat*	this study
SSY659	*pho8Δ::HIS3 pho13Δ::TRP YOP1-pho8Δ60::kan atg7Δ::LEU2 ego3Δ::nat*	this study
SSY660	*pho8Δ::HIS3 pho13Δ::TRP YOP1-pho8Δ60::kan atg7Δ::LEU2 vtc4Δ::nat*	this study

Strain	Relevant genotype	Source
SSY661	*pho8Δ::HIS3 pho13Δ::TRP YOP1-pho8Δ60::kan atg7Δ::LEU2 nvj1Δ::nat*	this study
SSY662	*pho8Δ::HIS3 pho13Δ::TRP YOP1-pho8Δ60::kan atg7Δ::LEU2 pep4Δ::nat*	this study
SSY671	*pho8Δ::HIS3 pho13Δ::TRP YOP1-pho8Δ60::kan atg7Δ::LEU2 vps4Δ::nat*	this study
SSY672	*pho8Δ::HIS3 pho13Δ::TRP YOP1-pho8Δ60::kan atg7Δ::LEU2 vps23Δ::nat*	this study
SSY673	*pho8Δ::HIS3 pho13Δ::TRP YOP1-pho8Δ60::kan atg7Δ::LEU2 vps24Δ::nat*	this study
SSY377	*opi1Δ::kan*	this study
SSY060	*pep4Δ::TRP1 prb1Δ::HIS3*	this study
SSY572	*pep4Δ::TRP1 prb1Δ::HIS3 opi1Δ::kan*	this study
SSY192	*pep4Δ::TRP1 prb1Δ::HIS3 atg7Δ::hph Yiplac-ssGFPHDEL-NatMX6*	this study
SSY602	*pep4Δ::TRP1 prb1Δ::HIS3 opi1Δ::kan atg7Δ::hph Yiplac-ssGFPHDEL-NatMX6*	this study
